# *Cistanches Herba*: A Neuropharmacology Review

**DOI:** 10.3389/fphar.2016.00289

**Published:** 2016-09-20

**Authors:** Caimei Gu, Xianying Yang, Linfang Huang

**Affiliations:** Institute of Medicinal Plant Development, Chinese Academy of Medical Sciences and Peking Union Medical CollegeBeijing, China

**Keywords:** *Cistanches Herba*, neuropharmacological effect, Alzheimer’s disease, Parkinson’s disease, review

## Abstract

*Cistanches Herba* (family Orobanchaceae), commonly known as “desert ginseng” or Rou Cong Rong, is a global genus and commonly used for its neuroprotective, immunomodulatory, anti-oxidative, kidney impotence, laxative, anti-inflammatory, hepatoprotective, anti-bacterial, anti-viral, and anti-tumor effects in traditional herbal formulations in North Africa, Arabic, and Asian countries. The major bioactive compound present in this plant is phenylethanoid glycosides. In recent years, there has been great important in scientific investigation of the neuropharmacological effects of the bioactive compounds. The *in vitro* and *in vivo* studies suggests these compounds demonstrate neuropharmacological activities against a wide range of complex nervous system diseases which occurs through different mechanisms include improving immunity function and kidney aging, anti-lipid peroxidation, scavenging free radical, inducing the activation of caspase-3 and caspase-8. This review aims to summaries the various neuropharmacological effects and mechanisms of *Cistanches Herba* extracts and related compounds, including its efficacy as a cure for Alzheimer’s disease and Parkinson’s disease with reference to the published literature. Which provides guidance for further research on the clinical application of *Cistanches Herba*.

## Introduction

**FIGURE 1:**
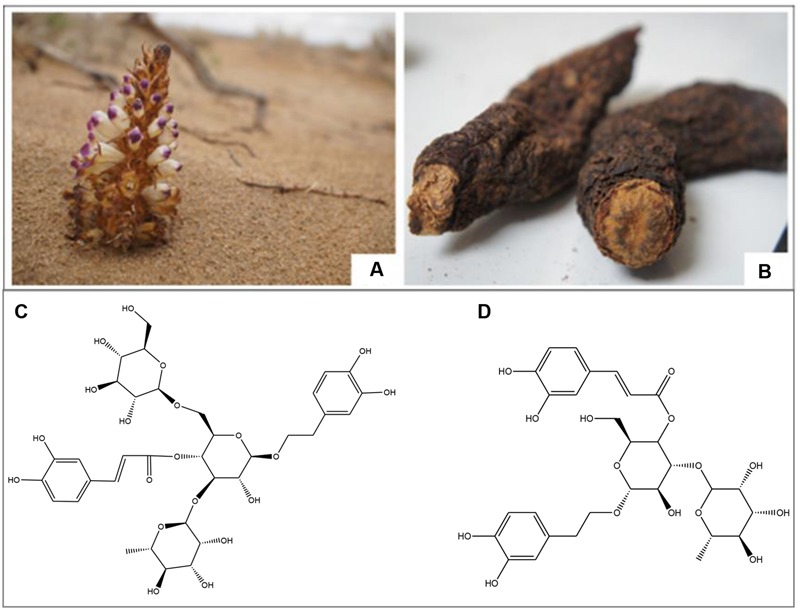
**The *Cistanches Herba* growed in Alashan, Inner Mongolia, China; and the structures of two important phenylethanoid glycosides in *Cistanche Herba*. (A)** The original *Cistanche deserticola* Y. C. Ma; **(B)** The dry *Cistanche deserticola* Y. C. Ma; **(C)** Echinacoside; **(D)** Verbascoside.

*Cistanches Herba*, the dried stem of Cistanches species *Cistanche deserticola* Y.C.Ma (**Figure 1**) and *Cistanche tubulosa* (Schenk) Wight, is recorded in the Chinese Pharmacopeia (Committee, 2015). Other non-official species, such as *C. sinensis* Beck and *C. salsa* (C. A. Mey) Beck, are also used as *Cistanches Herba* in certain regions of China due to resource shortage. *Cistanches Herba* is one of the most valuable herbal drugs in traditional Chinese medicine, which supplements kidney functions, boosts the essence of blood, and moistens the large intestines to free stool (Medicine, 2005). Therefore, it is called “desert ginseng” in China because of the excellent medicinal functions and nourishing effects (Wang et al., 2012). *Cistanches Herba*, a global genus of holoparasitic desert plant, which is primarily endemic in North Africa, Arabic, and Asian countries (Nan et al., 2013). The primary producing areas of *Cistanches Herba* in China are Inner Mongolia and the provinces of Xinjiang, Gansu and Qinghai.

Several chemical groups were isolated from *Cistanches Herba*, including PhGs (**Figure 1**), lignans, iridoids, and polysaccharides (Chen et al., 2013). Pharmacological studies demonstrated that *Cistanches Herba* exhibits neuroprotective, kidney impotence, laxative, anti-inflammatory, hepatoprotective, immunomodulatory, anti-oxidative, anti-bacterial, anti-viral, and anti-tumor effects (Hu and Feng, 2012). And our previous studies have distinguished *Cistanches Herba* from different geographic origins using a combination of DNA barcoding and UPLC-Q-TOF/MS technology.

The Consumer Price Indexdatabase of China reports that 58 drugs from 12 different groups, including glycosides of *Cistanche* capsules and compound *Cistanche* Yizhicapsules, are authorized for the treatment of AD. *Cistanches Herba* wine and tea are produced in Alashan, Inner Mongolia, China, which might assist in Runchang catharsis and enhance the immune, endocrine regulation, and anti-aging systems of the body. Boschnalosides used as a therapeutic agent in Japan to treat sexual dysfunction and amnesia, and echinacoside is used in healthcare products in the United States to improve immunity (Cheng et al., 2005).

Some researchers recently focused on the neuroprotective effects of *Cistanches Herba*, but these effects have not been studied thoroughly (**Table 1**). This review presents and analyzes recent developments in the neuropharmacology of *Cistanches Herba* and provides a reference for the further study and clinical application of this medicinal plant.

**Table 1:** The neuropharmacological effects of *Cistanches Herba*.

Pharmacological effects	Traditional and clinical uses	Extracts/ Compounds	Minimal active concentration/ Dose	Animal/ Cell line	*In vivo/ In vitro*	Duration	Control	References
Anti-Alzheimer’s disease	In Alzheimer’s disease–like mice model caused by reducing quinolinic acid: enhance learning and memory, protect the brain damage	Glycosides	62.5 mg/kg	Kunming mice	*In vivo*	17 days	Positive control: Duxil (8 mg/kg)	Liu et al., 2005
	In Alzheimer’s disease–like mice model caused by amyloid β-protein: enhance learning and memory	Glycosides	62.5 mg/kg	NIH mice	*In vivo*	17 days	Positive control: vitamin E (50 mg/kg)	Liu et al., 2006
	In Alzheimer’s disease–like mice model caused by AlCl_3_: improve learning and memory problems	Glycosides	62.5 mg/kg	Kunming mice	*In vivo*	20 days	Positive control: vitamin E (50 mg/kg)	Luo et al., 2007
	In Alzheimer’s disease–like mice model caused by amyloid β-protein: enhance learning and memory	Glycosides	25 mg/L	PC12 cell	*In vitro*	120 h	Positive control: Ginkgo laminae (40 mg/L)	Luo et al., 2010
	In Alzheimer’s disease–like rat model caused by amyloid β-protein: improve learning and memory	Glycosides	40 mg/kg	SD rats	*In vivo*	14 days	Positive control: vitamin E (50 mg/kg)	Luo et al., 2013
	In aging rat model caused by D-galactose: protect hepatic mitochondria	Alcohol extract	2 g/kg/d	Wistar rat	*In vivo*	6 weeks	Not mentioned	Xu et al., 2007
	In aging model caused by D-galactose: enhance oxidation resistance, improve mitochondrial energy metabolism	Polysaccharide	50 mg/kg	ICR mice	*In vivo*	4 weeks	Positive control: vitamin E (100 mg/mL)	Xu and Liu, 2008
	Protective effect of *Cistanche* polysaccharide on liver mitochondria in aging rats	Polysaccharide	2 g/kg/d	Wistar rat	*In vivo*	6 weeks	Not mentioned	Xu et al., 2008
	Antagonize free radical damage, enhance heart and brain telomerase activity and immune function in aging mice	Polysaccharide	25 mg/kg	ICR mice	*In vivo*	6 weeks	Normal control: normal saline	Zhang et al., 2011
	Protect cells from DNA damage, partly elucidating the mechanism of its effects and has potential anti-senescence activity	Acteoside and echinacoside	100 mg/kg	SAM-P8 mice and control SAM-R1 mice	*In vivo*	4weeks	Not mentioned	Zhang et al., 2014
	In aging rat model caused by D-galactose: enhance oxidation resistance, reduce liver mitochondrial oxidative damage	Water extract	40 mg/kg	ICR mice	*In vivo*	2 weeks	Normal control: normal saline	Zhang et al., 2008
	In aging rat model caused by D-galactose: antagonize free radical damage, enhance heart and brain telomerase activity and immune function	Echinacoside	1 μM	Cell line MRC-5	*In vitro*	48 h	Normal control: 250 μM H_2_O_2_	Xie et al., 2009
	Scavenge free radicals effectively and has protection against OH^∙-^induced DNA damage	Glycosides	50 μL	Not mentioned	*In vitro*	Not mentioned	Not mentioned	Wang et al., 2001
Anti-oxidative and Anti-apoptotic	Effect of Polysacchrides of *Cistanche deserticola* Y. C. Ma on Lipid Peroxide in Aging Mice	Polysacchrides	50 mg/kg	NIH mice	*In vivo*	7 weeks	Positive control: vitamin E (35 mg/kg)	Wu and Fu, 2004
	Protective effect of *Cistanche deserticola* on skeletal muscle oxidative injury in high intensity training rats	Alcohol extract	1 mg/g	SD rats	*In vivo*	4 weeks	Positive control: Oral Liquid of Rhodiola (0.5 Ml/100g)	Luo et al., 2012
	Rescues the SHSY5Y neuronal cells from TNFα-induced apoptosis	Echinacoside	1 mg/L	SH-SY5Y cell	*In vitro*	2 h	Not mentioned	Deng et al., 2005
Improve learning and memory	Effects of Cistanchis glycosides on the Learning and Memory of Kidney Yang Deficiency Mice	Glycosides	100 mg/kg	Kunming mice	*In vivo*	30 days	Positive control: Jing guishen qi pill (1.5 g/kg)	Gao et al., 2005
	The effect of phenylethanoid glycosides of the *Cistanche deserticola* on scopolamine-induced impairment of learning memory in mice	Phenylethanoid glycosides	10 mg/kg	Kunming mice	*In vivo*	Not mentioned	Normal control: normal saline	Li, 2011
	Enhance the ability of learning and memorizing	Phenylethanoid glycosides	50 mg/kg	Kunming mice	*In vivo*	30 days	Normal control: normal saline	Liu et al., 2011
	Enhances learning and memory by inducing nerve growth factor	Glycosides	10 μg/mL, 10 μL	PC12 cell line	*In vitro*	48 h	Positive control: nerve growth factor (50 μg/mL)	Choi et al., 2011
	Improve the learning and memory ability	Glycosides	2.5 mg/kg/d	Wistar rat	*In vivo*	14 days	Positive control: Oxiracetam (450 mg/kg/d)	Feng et al., 2013
Anti-Parkinson’s disease	Improves the behavioral and neurochemical outcomes in MPTP mice model of Parkinson’s disease and inhibits caspase-3 and caspase-8 activation in cerebellar granule neurons	Echinacoside	5 mg/kg	C57BL/6 mice	*In vivo*	15 days	Positive control: amantadine (40 mg/kg)	Geng et al., 2007
	Protect neurons	Acteoside	25 mg/L	Wistar rat	*In vivo*	12 h	Not mentioned	Pu et al., 2001
	Protecting effect of *Cistanche* extracts on MPP^+^ induced injury of the Parkinson’s disease cell model	Extract	2 μg	SH-SY5Y cell	*In vitro*	48 h	Not mentioned	Wang et al., 2007
	Reduce 6-OHDA-induced ROS production in PC12 cells	Echinacoside	0.1 μM	PC12 cells	*In vitro*	24 h	Not mentioned	Wang Y.H. et al., 2015

## Related Literature Analysis

**FIGURE 2:**
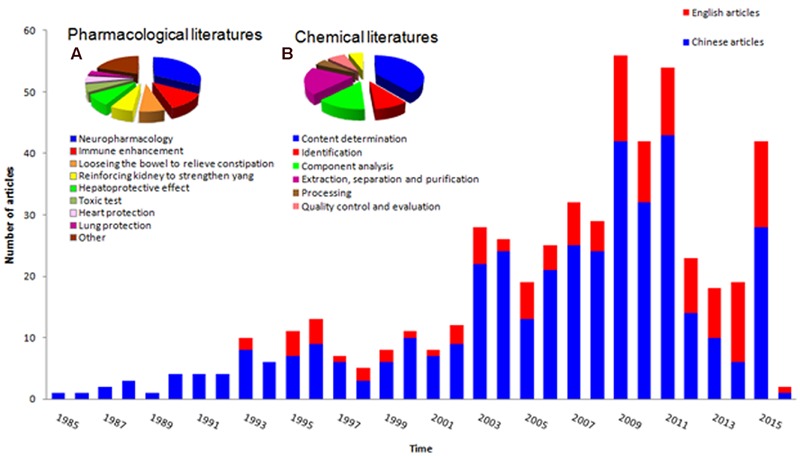
**Statistical analysis for published literatures of *Cistanches Herba*. (A)** Pharmaceutical literatures analysis of *Cistanches Herba*; **(B)** Chemical literatures analysis of *Cistanches Herba*.

*Cistanches Herba* medicines have a long history of practical use, but scientists worldwide only began to disclose their chemical composition in the1980s. **Figure 2** shows an analysis of the related literature. The cumulative histogram shows the number of studies increased over time, and the Chinese literature occupies the greatest proportion, which reveals the potential research value of *Cistanches Herba*. **Figure 2A** shows that the neuropharmacology related literature occupies the largest proportion of the nine areas of pharmacology, and this topic has become the most important area for research. **Figure 2B** exhibits the chemical research diversity of *Cistanches Herba*, with a substantial proportion of research on content determination. Further research may focus on neuropharmacology and component content.

## Traditional Use and Ethnopharmacology

*Cistanches Herba* has a long history as a medicinal plant in China and Japan because of its wide spectrum of pharmacological activities. It is commonly called *Rou Cong Rong* in Chinese, and it was first listed medicinal use as a tonic agent in the Chinese Materia Medica *Shen Nong’s Herbal Classic* (Estern Han Dynasty) 2000 years ago, and later recorded in *Yao Xing Lun* in 1590. The Compendium of Materia Medica (*Ben Cao Gang Mu*, 1619) documented that *Cistanches Herba* invigorated the kidney to treat kidney deficiencies and geriatric constipation strengthened and nourished marrow and essence, protected semen, and moistened dryness to relax the bowels. These properties were also written in *Ben Cao Hui Yan* in 1619. A total of 200 medicinal books recorded the pharmacodynamics and use of *Cistanches Herba* in Chinese history. *Cistanches Herba* ranks first in Chinese traditional medicine to strengthen prescriptions, which ranks second in anti-aging prescriptions at the same time, behind *Panax ginseng* in past dynasties. Modern pharmacological investigations demonstrated that *Cistanches Herba* was used as a kidney-yang reinforcing Chinese medicinal tonic, but it is also exhibits anti-aging, improves memory, and enhances immunity effects (**Table 1**), which indicate that extracts or constituents from *Cistanches Herba* have a promising future for the treatment of diseases, particularly nervous system disorders. However, systematic data on the pharmacological activities of this agent is lacking. It is urgent and important to study the pharmacological effects and mechanisms of *Cistanches Herba* deeply in the future.

## Pharmacology

### Anti-aging Properties

Aging is an inevitable process of life. This process involves a series of degenerative changes in tissues and organ functions with advancing age. Studies on aging and anti-aging medicines have made significant progress in recent years. Therefore, anti-aging drugs are a current and prominent issue in gerontology. The aging process reflects a confluence of *in vivo* and *in vitro* factors. Aging is closely related to type 2 diabetes, atherosclerosis, and AD. Aging is also related to the decreased regeneration of cells, viscera deficiency, increased free radicals, body poisoning, and lack of rhythm when eating (Lopez-Otin et al., 2013). Aging is an inevitable process, but delaying this process is now possible.

Several historical Chinese herbal pharmacopeias describe that *Cistanches Herba* possesses anti-aging properties. PhGs and oligosaccharides are two types compounds isolated from *Cistanches Herba* that are the main active ingredients of this plant. *In vivo* studies established an aging mouse model caused by D-galactose. The mice were divided into normal control, model, Vitamin E and total glycoside groups, and all groups received different doses of various materials. The results suggested that the glycosides exhibited protective effects on the hippocampal ultrastructure, and glycosides may play a role in the delay of aging and the prevention and treatment of senile dementia via anti-oxidation (Wang X. et al., 2015). Xu et al. investigated the protective effect of *Cistanches Herba* alcohol extract on hepatic mitochondria and established an aging rat model caused by D-galactose. Rats were administered *Cistanches Herba* alcohol extract for 6 weeks. The results indicated that Ca^2+^-ATP enzyme activity was enhanced, and the MDA content in the hepatic mitochondria was reduced. These results further suggested that the *Cistanches Herba* alcohol extract effectively protected hepatic mitochondria in the D-galactose aging rat model (Xu et al., 2007). Xu and Liu (2008) examined the anti-aging effect of PhGs isolated from *Cistanches Herba*. The results confirmed that the PhGs improved learning and memory, exhibited antioxidant activity, and boosted the immune system. The results also demonstrated that the PhGs exhibited anti-aging effects via enhancement of anti-oxidation. The mechanism may be related to the free radical scavenging ability of PhGs. Polysaccharides of *Cistanches Herba* exhibit the same function as PhGs on anti-aging (Xu et al., 2008; Zhang et al., 2011). Zhang et al. (2014) also investigated a *Cistanches Herba* extraction 2014 and found that this extract extended life span. The results of studies on echinacoside and acteoside suggest that these components exhibit positive anti-aging effects (Zhang et al., 2008; Xie et al., 2009). Many studies of anti-aging involve *Cistanches Herba*, but these works are limited because the anti-aging mechanism is not known. There are three possible pathways to anti-aging, including improving immunity function and kidney aging, anti-lipid peroxidation. Immune theory of aging said that the decline of immune function is closely related to the aging organism. Thus, the immune function of the body can indirectly reflect the aging organism in a certain extent. The raised index of thymus and spleen, increased content of IFN-γin serum and decreased content of IL-6, increased capacity of peritoneal macrophage phagocytic and lymphocyte proliferation response always can improve the immunity aging, and then delay the organism aging. The expression of p53 from human fibroblastic cell down-regulated significantly in a dose dependent manner after treatment with echinacoside, and which may be correlated with the up-regulation of SIRT1. The PhGs can scavenge different ROS, including. $O_{2}^{–}$, H_2_O_2_ and ⋅OH, effectively and protect DNA damage through scavenging ⋅OH. In addition, the PhGs also can increase the content of RNS- NO, and then reduce the lipid peroxidation. Therefore, the real effective components of *Cistanches Herba* and what a role in anti-aging are important and appealing future research directions.

### Anti-oxidative and Anti-apoptotic Activity

*Cistanches Herba* exhibits anti-oxidative, free radical-scavenging and anti-apoptotic activity via different mechanisms. Recent studies demonstrated the anti-oxidant activity of *Cistanches Herba*, particularly in the clearing of all types of free radicals *in vivo* and *in vitro*, improvement in the activity of anti-oxidant enzymes in the body, and inhibition of the formation of lipid peroxide, MDA, and brown fat (Wang et al., 2001; Wu and Fu, 2004; Luo et al., 2012; Song, 2013). Current studies demonstrated that cell apoptosis or programmed cell death is determined by heredity and is related to oxidation (Martin, 2011). Deng used the MTT assay to examine cell survival rate, agarose gel electrophoresis of DNA, and flow cytometry to detect cell apoptosis. The results suggested that the echinacoside extracted from *Cistanches Herba* exhibited protective effects on TNFα-induced SH-SY5Y cell apoptosis (Deng et al., 2005). Nerve cell protection exhibits a close connection with the reduction of active oxygen levels in cells, inhibition of caspasc-3 activity and maintenance of a high-energy state of mitochondrial membrane potential. Bao et al. (2010) investigated an extract of *Cistanche tubulosa* and discussed its anti-oxidant ability. These researchers conducted an *in vitro* study to compare the anti-oxidative properties of methanol and ethanol extracts. The results suggested that the two extracts exhibited high anti-oxidant ability, and 70% ethanol was the best extraction agent of *C. tubulosa* to ensure improved anti-oxidant activity (Bao et al., 2010). The PhGs from *Cistanches Herba* are considered the effective ingredients for anti-oxidative and anti-apoptotic activity in recent studies. The anti-oxidant mechanism is mainly related to the radical-scavenging activity. PhGs compounds, which are mostly provided with different amounts of phenolic hydroxyl, can be used as hydrogen donor to reductive radicals, and then reach to the purpose of radical scavenging. The *Cistanches Herba* scavenge the free radicals mainly in two ways, including directly involving in the removal of free radicals or blocking their production and regulating the anti-oxidant enzymes related to the free radical metabolism *in vivo*, such as SOD, CAT and GPX (Ko and Leung, 2007). For another, the Glycosides of *Cistanches Herba* can prevent the apoptosis of cerebellar granule neurons by inhibiting the activities of caspase-3 and caspase-8. Therefore, the good oxidation resistance and anti-aging ability of *Cistanches Herba* may be applied to cosmetics. This application may be a new research direction in the future.

### Learning and Memory Enhancement

Learning and memory are advanced functions of the brain, and these functions are important factors in determining intelligence. Learning and memory impairment is a common symptom in different types of encephalopathy, such as attention deficit and hyperactivity disorder in childhood, adolescent chorea, lobar atrophy disease, neurosis, senile cerebral arteriosclerosis, and dementia. Medicinal research demonstrated that the impairment of learning and memory is closely related to the impairment of synaptic transmission in the brain and the metabolism of neurotransmitters, other substances, and energy in the brain (Chen, 1993). Modern pharmacological studies determined that *Cistanches Herba* significantly improves learning and memory, and PhGs are the active chemical ingredients of this effect.

Traditional Chinese medicine shows that learning and memory dysfunction exists in the Yang deficiency model of the spleen and kidney. Therefore, these two models are more suitable for the study of tonics in traditional Chinese medicine. Gao et al. (2005) examined the effects of *Cistanches Herba* glycosides on the learning and memory of kidney Yang deficiency mice. The results of this study demonstrated that the Yang deficiency symptoms of each dose group improved, and the number of animal deaths decreased significantly. However, the jumping latency of each dose group after hydrocortisone administration was significantly prolonged, and the number of errors during a 5-min period was reduced. Therefore, glycosides improved the learning and memory of kidney Yang deficiency mice induced by hydrocortisone and reduced the death rate of these animals (Gao et al., 2005). The current researchers established a scopolamine-induced learning and memory impairment mouse model to investigate the effects of the PhGs of *Cistanches Herba*. The results demonstrated that the PhGs of *Cistanches Herba* enhanced learning and memory (Li, 2011; Liu et al., 2011). Choi et al. (2011) also demonstrated that *Cistanches Herba* enhanced learning and memory via the induction of nerve growth factor. Vary factors related to cerebrovascular disease induce vascular dementia. This condition is an acquired intelligence-damaging syndrome of cognitive impairment, which is a primary type of senile dementia. Ischemic cerebrovascular disease occurs frequently in many cerebrovascular diseases induced by vascular dementia. Traditional medicine and modern pharmacology demonstrated that PhGs play an active role in neuroprotection (Feng et al., 2013; Liu et al., 2013; Zhu et al., 2013; Zhang, 2014). The reason why *Cistanches Herba* extract can improve learning and memory is partly due to neuronal cell differentiation, neurite outgrowth and presynaptic formation promoted. *Cistanches Herba* also improved cognitive behavior related to memory ability. Therefore, *Cistanches Herba* is a potential candidate for cognitive enhancement owing to its action as a nerve growth factor modulator. However, extensive research is necessary to discover the neuroprotective mechanism deeply. Further studies to determine the specific type of PhGs are expected to play a leading role in improving learning and addressing memory impairment.

## Anti-Neurodegenerative Diseases

### Anti-Alzheimer’s Disease

**FIGURE 3:**
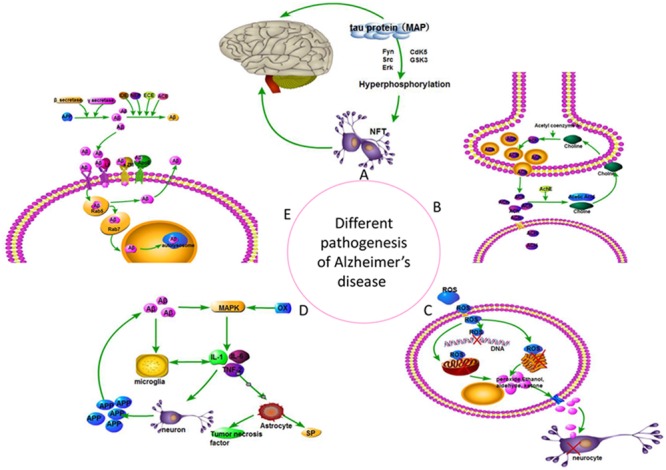
**Different pathogenesis hypothesis of Alzheimer’s disease. (A)** Tau hypothesis; **(B)** Cholinergic hypothesis; **(C)** Free radical damage hypothesis; **(D)** Inflammation hypothesis; **(E)** Amyloid cascade hypothesis.

Alois Alzheimer originally described AD in 1906 based on his observations of amyloid plaques, neurofibrillary tangles, and vascular anomalies during the autopsy of August Deter, a patient who died with severe cognitive defects. AD is a progressive neurodegenerative disorder that affected more than 30 million people worldwide in 2010 (Williams et al., 2011). The pathogenesis of this disease is complex and not completely understood. However, several potential causative factors were proposed, including cholinergic neurons, Aβ toxicity, tau protein, insulin, and free radical damage. Considerable attention was devoted recently to inflammation, insulin, oxidative imbalance, and gene mutation hypotheses (Verma et al., 2016) (**Figure 3**). Numerous drugs reduce the symptoms of AD, but no cure has been developed. Drugs that treat AD currently include six major classes: cholinesterase inhibitors, NMDA receptor antagonists, neurotrophic factors, drugs that promote nerve cell metabolism, neuroprotective agents, and traditional Chinese medicine (Deardorff and Grossberg, 2016).

Studies of traditional Chinese medicine suggest that *Cistanches Herba* has positive effects on the treatment of AD. An *in vitro* study of the effects of *Cistanches Herba* in quinolinic acid-reduced AD mouse model determined that the content of MDA and calciumin brain tissue was reduced, the activity of SOD and GSH-Px improved, and the activity of acetylcholinesterase E and the apoptosis rate of brain cells was depressed. These results suggest that the glycosides of *Cistanches Herba* enhance learning and memory and protect the brain from damage. The mechanism may be related to the enhanced free removal of enzyme activity, reduced lipid oxidation reaction, lower brain tissue calcium content and the inhibition of apoptosis of brain cells induced by QA (Liu et al., 2005). Liu et al. established an AD mouse model in 2006 using a one-time microinjection of β-AP_25-35_, which reduced β-AP deposition in the brain. Repeated administration of glycosides of *Cistanche* for 10 days reduced the MDA content and apoptosis rate of brain cells, improved SOD and GSH-Px, weakened Bax expression, and enhanced Bc-l2 expression. Bax weakening and enhanced Bc-l2 may induce the enhanced free removal of enzyme activity, reduced lipid oxidation reaction and inhibition of apoptosis of brain cells (Liu et al., 2006). Luo et al. also used the AD mouse model induced by the subcutaneous injection of aluminum chloride and demonstrated that *Cistanches Herba* enhanced learning and memory. The mechanism is closely connected with anti-oxidation (Luo et al., 2007). The previously mentioned models of AD were established using different methods. However, the results consistently illustrated that *Cistanches Herba* glycosides significantly improved learning and memory impairments in the brain. Studies also suggested that the underlying mechanism of these glycosides related to anti-oxidative actions, enhancement of free radical scavenging activity and inhibition of brain cell apoptosis. Luo, Gao and Wu et al. (Luo et al., 2010, 2013; Ying et al., 2014) conducted similar experiments and revealed that various components of *Cistanches Herba*, including glycosides, extract, echinacoside, and acteoside, exhibit active anti-AD effects to varying degrees (Supplementary Table S1). The mechanisms related to anti-AD including free radical scavenging activity enhancement, lipid peroxidation improvement, apoptosis inhibition, the production of active oxygen decrease and apoptotic signal pathway regulating. Additionally, previous research have studied the mechanism from the point of proteomics that the echinacoside can reduce the over-expression of biliverdin reductase B, which suggest that the anti-oxidative activity of echinacoside can reduce the increasing of biliverdin reductase B induced by oxidative stress and protect the dopaminergic neurons from oxidative stress injury. However, the real cause of AD and the resistance mechanism of *Cistanches Herba* need us spend more efforts to explore.

### Anti-Parkinson’s Disease

Parkinson’s disease is a neurodegenerative disorder that is characterized by the selective degeneration of dopaminergic neurons in the substantia nigra pars compacta and a consequent reduction in striatal dopamine levels in the brain. PD is one of the most common diseases in the elderly. The clinical manifestations of this disease include hypokinesia, skeletal muscle tension, and resting muscle tremor. British doctor James Ba Jinsen (Parkinson) first described this unusual group of symptoms in 1817, and the disorder was eventually named after him. PD is second only to AD as the most common degenerative disease, which exhibits an incidence rate of 1–2% of people aged 65 years and older and increases with advancing age. Neuropathological hallmarks of PD were described extensively, but its etiology is not defined. Genetic and environmental factors may play a pivotal role in the causes of PD. Previous studies demonstrated that oxidative stress, a cellular dysfunction between the production and scavenging of free radicals, was the primary mechanism of the associated with neuronal death (Liu et al., 2014; Wen and Wang, 2014; Huang, 2015; Lin et al., 2015; Ma, 2015; Ou et al., 2015; Peng and Bai, 2015; Wen and Xu, 2015). The most extensively used of PD mouse model is produced via the systemic administration of the neurotoxin MPTP. MPTP is switched on by monoamine oxidase B within non-dopaminergic cells, predominantly glial cells, to its active metabolite 1-methyl-4-phenylpyridinium (MPP^+^), which enters dopaminergic neurons through the dopamine transporter. The accumulation of MPP^+^ in the substantia nigra pars compacta neurons inhibits complex of the mitochondrial electron transport chain, which ultimately leads to cell death (Geng et al., 2007). The *in vivo* study demonstrated that campneoide and tubuloside B of *Cistanches Herba* protected neurons from MPP^+^-induced apoptosis (Pu et al., 2001; Sheng et al., 2002). Geng et al. (2004) investigated the neuroprotective effects of PhGs from *Cistanches salsa* against MPTP-induced dopaminergic toxicity in C57 mice and demonstrated that echinacoside improved the behavioral defects of PD mice. The PhGs could be attractive candidates against some typical neurological diseases, such as PD. Chen et al. investigated the echinacoside of *Cistanches salsa* and demonstrated that this natural phenol may be useful in PD prevention and treatment (Chen et al., 2007).

Parkinson’s disease has received significant attention, but this disease is not fully understood. Cure is not available because of the complex etiology and pathogenesis of PD, and the therapeutic tool for this disease is primarily drug treatment. However, long-term drug use likely results in serious side effects. Therefore, a treatment that combines Chinese and Western medicines is important. Further research on PD pathogenesis and therapeutic tool is necessary to reduce the side effects of drugs and obtain good treatment effects.

## Toxicology

*Cistanches Herba* was commonly considered a safe traditional medicine in China for 1000 of years (*Shen Nong’s Herbal Classic*). Common adverse clinical reactions of preparations of *Cistanches Herba* are mild and include nausea and vomiting, abdominal pain, and dizziness. However, recent investigations indicated that extracts of *Cistanches Herba* exhibit no obvious toxicity (Peng et al., 2011; Jiang et al., 2013; Huang et al., 2014; Qin et al., 2015). Toxicology may be a new direction of future research.

## Discussion and Future Perspectives

*Cistanches Herba* has been the increasing interest subject in recent years, and many traditional uses have been validated by pharmacological studies. Previous animal investigations and *in vitro* studies of plant preparations revealed that *Cistanches Herba* possesses a significant neuroprotective effect. *Cistanches Herba* extract and its constituents not only exhibit positive activities in AD and PD treatments but also against other diseases and conditions, such as aging, and learning and memory impairments. These results support the considerably high value of *Cistanches Herba*, which plays a significant role in clinical application and provides a potential basis for new drugs against nervous system diseases. Pharmacological analysis of *Cistanches Herba* extracts and related compounds also provided new insights into the treatment of nervous system diseases.

Previous researches have investigated the pharmacokinetics and bioavailability of PhGs. In Jia’s study (Jia et al., 2006), the echinacoside in rat serum was tested in four kinds situation. Results showed that the echinacoside in biosamples was susceptible to degradation at a higher temperature during the whole process and the operation must be carried out at lower temperature. The absorption of echinacoside was extaemely fast (T_max,_ 15 min) in rats after intragastric administration (100 mg/kg). However, the serum concentration maximum was very low (C_max_, 612.2 ± 320.4 ng/mL). The elimination was fast after intragastric administration (T_1/2_, 74.4min). And on the other hand, the distribution and elimination of echinacoside were extremely fast in rats (t_1/2α,_ 12.4min; t_1/2β_, 41.0min) after intravenous administration. The bioavailability of echinacoside in rat was very low, which was consistent with the result in Matthias’ report (Matthias et al., 2004). Wu et al. (2006) explored the pharmacokinetics of acteoside in *Cistanches Herba* in rats. The results showed that acteoside was evenly distributed in brain tissues which was about 0.45–0.68% of that in plasma after 15 min of acteoside intragastric administration. The bioavailability of acteoside and echinacoside were both very low, which always can through the blood brain barrier and reach the brain. The wide dosage range listed in tables may be associated with the low bioavailability. To improve the bioavailability of PhGs, numerous approaches may be undertaken, including the use of liposomal PhGs, PhGs nanoparticles, the use of PhGs phospholipid complex and the use of structural analogs of PhGs. Further study needs to be carried out to increase the bioavailability and elucidate the human absorption mechanism.

In this review we can see that most neuropharmacological effects have closely relationships with the antioxidant activity of *Cistanches Herba*. Thus the further study of antioxidant is needed urgently. The development and discovery of a new drug from *Cistanches Herba* requires much more detailed investigations of its pharmacological mechanism, pharmacokinetic and clinical use, particularly at the molecular level, despite continued progress on various aspects of this plant. Further studies on *Cistanches Herba* will address an urgent medical need through the development of efficacious treatments for nervous system disease.

## Author Contributions

CG and LH designed the study. XY collected the data of pathogenesis of Alzheimer’s disease and drew the **Figure 3**. CG wrote the manuscript and made the other figures and tables. XY and LH added the helpful discussions. CG, XY, and LH edited the manuscript, figures and tables.

## Conflict of Interest Statement

The authors declare that the research was conducted in the absence of any commercial or financial relationships that could be construed as a potential conflict of interest.

## Abbreviations

Aβ: β-amyloid
AD: Alzheimer’s disease
GSH-Px: glutathione peroxidase
MDA: malondialdehyde
PD: Parkinson’s disease
PhGs: phenylethanoid glycosides
RNS: reactive nitrogen species
ROS: reactive oxygen species
SOD: superoxide dismutase
