# Supplementary material for: Cistanches Herba: A Neuropharmacology Review
Source: Front Pharmacol. 2016 Sep 20;7:289. doi: 10.3389/fphar.2016.00289 (PMC5028387; doi:10.3389/fphar.2016.00289)
Supplement: Supplementary file 1 [file Table_1.DOCX]

**Supplementary Table 1**. The pharmacological effects of Cistanches Herba.

| Time | Title | Traditional and clinical uses | Extract type | Animal/ Cell line | In vivo/ In vitro | Dose rang | Duration | Control |
| --- | --- | --- | --- | --- | --- | --- | --- | --- |
| 1988 | Effects of *Cistanche deserticola* on immune function of mice | Enhancing  immunity | Aqueous extract | Kunming Mice | In vivo | 50-100mg/kg | 72h | Blank control 0.9% saline |
| 1990 | Research on anti-aging effects of *Cistanche deserticola* | Anti-aging | 95% Ethnol extract | Kunming Mice | In vivo | 2.59-51.259g/kg | 18d | Blank control distilled water |
| 1990 | Overview of *Cistanche* component and pharmacology | Review |  |  |  |  |  |  |
| 1991 | Effects on drosophila melanogaster longevity of *C. deserticola* and framboise | Anti-aging | Aqueous extract | Drosophila elanogaster | In vivo | 5%-10% |  |  |
| 1992 | Research on anti-atherosclerosis of *Cistanche deserticola* | Anti-  atherosclerosis | Aqueous extract | Rabbit | In vitro | 500µg/mL | 96h | Positive control vitamin E |
| 1992 | Laxative effect of *Cistanche deserticola* | Loosening bowel to relieve constipation | Water  decoction | Kunming Mice | In vivo | 0.6µL/mouse | 5h | Blank control distilled water |
| 1993 | Pharmacological activity and composition of the ethyl acetate extract of *Cistanche* phelypaea |  | Acteoside | Kunming Mice | In vivo |  |  |  |
| 1993 | Research on Yang animal kidneys induced by hormone and adrenal of *Cistanche* alcohol extract | Kidney protection | Ethnol extract | Kunming Mice | In vivo | 15mg/ mouse/d | 15d | Negative control Hydrocortisone Acetate |
| 1993 | Effect of *Cistanche deserticola* on lipid peroxidation and superoxide dismutase in rat | Antioxidant | Ethnol extract | Sprague-Dawley rats | In vitro | 100-200mg/kg/d | 15d | Blank control 0.9% saline |
| 1993 | Comparison of laxative effect between crude drug and processed products | Loosening bowel to relieve constipation |  | Kunming Mice | In vivo | 0.5mL/ mouse |  | Blank control distilled water |
| 1994 | Comparison of invigorating the kidney and strengthening Yang among three *Cistanche* species | Reinforcing  kidney to  strengthen yang | Water  decoction | Kunming Mice | In vivo | 0.03mL/g | 15d |  |
| 1995 | The effects of traditional Chinese medicine *Cistanche* species on the immune function and lipid peroxidation | Enhancing  immunity | Ethyl acetate  extract | Kunming Mice | In vitro | 1-5g/L | 24h | Blank control 0.9% saline |
| 1995 | Comparison of chemical composition and pharmacological effects between *Cistanche deserticola* and *Cistanche salsa* |  | Phenylethanoid glycosides | Sprague-Dawley rats | In vivo | 1.20-3.80g/kg | 9d | Positive control phenol-red |
| 1995 | Morphological changes of peripheral blood corpuscles of radiated rats feeded with *Cistanche* | Enhancing  immunity | Cistanche glycosides | Kunming Mice | In vivo | 125-250mg/mL | 15d | Blank control 0.9% saline |
| 1996 | Antioxidative effects of phenylethanoids from *Cistanche deserticola* | Antioxidant | Phenylethanoid glycosides | rats | In vivo |  |  |  |
| 1996 | Comparison of invigorating the kidney and strengthening Yang among three *Cistanche* species | Reinforcing  kidney to  strengthen yang | Water  decoction | Kunming Mice | In vivo | 0.5-1.0mL/d | 14d | Positive control Guilingji yang medicine, Negative control hydrocortisone |
| 1996 | Comparison between *C. deserticola* Y. C. Ma and *C. deserticola tubulosa* on some pharmacological actions |  | Water  decoction | Wistar rats | In vivo | 2mg/kg | 12d | Positive control testosterone propionate, Blank control 0.9% saline |
| 1997 | Antilipid peroxidation and antiradiative action of glycosides in Herba *Cistanche* | Antioxidant | Cistanche glycosides | Kunming Mice | In vivo | 62.5-250mg/kg/d | 18d | Blank control 0.9% saline |
| 1997 | The regulation efforts of Herba *Cistanche* polysaccharides on immunological functions of liver- Qi stagnation and spleen deficiency induced by CCl_4_ in mice | Hepatoprotection | Polysaccharides | Kunming Mice | In vivo | 200-400mg/kg/d | 14d | Negative control carbon tetrachloride, Blank control 0.9% saline |
| 1997 | Pharmacological action of *Cistanche deserticola* polysaccharides on tumor bearing mice | Antitumor | Polysaccharides | Kunming Mice | In vivo | 200-400mg/kg/d | 10d | Blank control 0.9% saline |
| 1998 | Studies on the sedative effect of Cistanche deserticola | Sedation | Ethnol extract | Kunming Mice | In vivo | 0.1-1.0g/kg | 1h | Blank control 0.9% saline |
| 1998 | Hepatoprotective activity of phenylethanoids from Cistanche deserticola | Antioxidant | Phenylethanoid glycosides | Rabbit | In vivo |  |  |  |
| 1998 | Purification of polysaccharides of *C. deserticola* Y. C. Ma and its immunomodulatory effects on T cell function | Enhancing  immunity | Polysaccharides | Kunming Mice | In vitro | 100µL/ mouse |  |  |
| 1998 | Anti-oxidative action of the glycosides of Cistanche in the tissues of mice | Antioxidant | Cistanche glycosides | Kunming Mice | In vivo | 62.5-250mg/kg/d | 30d | Blank control 0.9% saline |
| 1998 | Effect of *C. deserticola* Y. C. Ma on lipid peroxide content in the brain and liver of the elderly rats | Antioxidant | Water  decoction | Kunming Mice | In vivo | 100-200mg/kg | 28d | Blank control 0.9% saline |
| 1999 | (2E,6R)-8-hydroxy-2,6-dimethyl-2-octenoic acid, a novel anti-osteoporotic monoterpene, isolated from *Cistanche salsa* | Anti-osteoporosis | Ethnol extract | Kunming Mice | In vivo | 16mg/mouse/d | 4 weeks |  |
| 1999 | The protection on myocardial ischemia of *Cistanche* glycosides | Protect ischemic myocardium | Cistanche glycosides | Wistar rats | In vivo | 125-250mg/kg | 240min | Positive control salviae miltiorrhizae |
| 1999 | Effect of Herba *Cistanche* on micturition in urethan anesthetized rats | Improving the  function of  urination | C. deserticola extract | Wistar rats | In vivo | 1.0-3.0g/kg | 1h |  |
| 1999 | A experiment study on the immune pharmacologic effects of polysaccharides of Herba *Cistanche* | Enhancing  immunity | Polysaccharides | Kunming Mice | In vivo | 0.2-200mg/kg/d | 7d |  |
| 1999 | Comparison on moistening the intestines and laxation of Herba *Cistanches* | Loosening bowel to relieve constipation | Water  decoction | Kunming Mice | In vivo | 0.5-1.0mL /d | 10d | Positive control Maren Runchang pill |
| 2000 | Inhibition of nitric oxide by phenylethanoids in activated macrophages | Anti-inflammatory | Phenylethanoid glycosides | Kunming Mice | In vivo |  | 24h |  |
| 2000 | Protective effects of glycosides of *Cistanche* on hematopoietic system of ^60^Coγ-ray irradiated mice | Protecting the hematopoietic system | Cistanche glycosides | NIH Mice | In vivo | 62.5-250mg/kg | 21d | Positive control astragalus injection |
| 2000 | Effects of glycosides of *Cistanche* on lipid peroxidation in hemorrhagic shock/ reperfusion injury | Antioxidant | Cistanche glycosides | Rabbit | In vivo | 250mg/kg | 140min |  |
| 2000 | Effect of *Cistanche* polysaccharides on rats with spleen deficiency syndrome |  | Polysaccharides | Sparague-Dawley rats | In vivo | 150-300mg/100g | 7d | Negative control rhubarb extract |
| 2000 | A morphological and histochemisty study of effects of *Cistanche deserticola* on rat testis and epidermis | Reinforcing  kidney to  strengthen yang | Water  decoction | Kunming Mice | In vivo | 4-8g/kg | 21d | Blank control 0.9% saline |
| 2000 | Protective effects of glycosides of *Cistanche* against isoprenaline-induced myocardial damage of mice | Protect ischemic myocardium, Antioxidant | Cistanche glycosides | NIH Mice | In vivo | 125-500mg/kg | 7d | Positive control propranolol |
| 2001 | Purification of phenylethanoids from Brandisia hancei and the anti proliferative effects on aortic smooth muscle | Anti-atherosclerosis | Phenylethanoid glycosides | Wistar rats | In vitro |  | 48h |  |
| 2001 | Protective effects of glycosides of *Cistanche* glycosides on ultrastructure of sensitive organs of ^60^Co irradiated mice | Protect sensitive organ ultrastructure | Cistanche glycosides | NIH Mice | In vivo | 62.5mg/kg | 11d | Blank control 0.9% saline |
| 2001 | Metabolic regulation of phenylethanoid glycosides from Herba *Cistanches* in dogs gastrointestine | Loosening bowel to relieve constipation | Phenylethanoid glycosides | Beagle dog | In vivo | 3g/kg | 24h | Blank control 0.9% saline |
| 2001 | Protective effects of glycosides of *Cistanche* on immune function of ^60^Coγ-ray irradiated mice | Enhancing  immunity | Cistanche glycosides | NIH Mice | In vivo | 31.25-125mL/kg | 30d | Positive control astragalus injection |
| 2001 | Effect of *Cistanche deserticola* on urinary and deficiency shen- yang rats | Improving the  function of  urination | C. deserticola extract | Wistar rats | In vivo | 0.2mL/100g | 24h | Negative control hydrocortisone |
| 2001 | Effects of *Cistanche deserticola* polysaccharides on the constitution of protein and anti-oxidative capacity of lune in aging mice | Antioxidant , Anti-aging | Polysaccharides | ICR Mice | In vivo | 50-100mg/kg/d | 40d | Blank control 0.9% saline |
| 2002 | Anti-nociceptive and anti-inflammatory activity caused by *Cistanche deserticola* in rodents | Anti-inflammatory | Crude drug | ICR mice | In vivo | 0.03-1g/kg | 240min | Positive control naloxone |
| 2002 | Tubuloside B from *Cistanche salsa* rescues the PC12 neuronal cells from 1-methyl-4-phenylpyridinium ion-induced apoptosis and oxidative stress | Antiapoptotic | Tubuloside B | rattus norvegicus | In vitro |  |  |  |
| 2002 | Immunomodulatory effects of polysaccharides of *C. deserticola* Y. C. Ma | Enhancing  immunity | Polysaccharides | ICR Mice | In vivo | 50-100mg/kg/d | 40d | Blank control 0.9% saline |
| 2002 | The change of functions and hypermicro- instructure in lung in experimental aging mice and effect of *Cistanche deserticola* polysaccharides on the change |  | Polysaccharides | ICR Mice | In vivo | 50-100mg/kg | 42d | Blank control 0.9% saline |
| 2003 | Acteoside from *Cistanche* salsa inhibits apoptosis by 1-methyl-4-phenylpyridinium ion in cerebellar granule neurons | Antiapoptotic |  |  | In vitro | 12.5-50µm | 72h |  |
| 2003 | Protective efforts of glycosides of *Cistanche* on cerebral ischemia-reperfusion injuries in awake mice | Protection of cerebral ischemia reperfusion injury | Cistanche glycosides | Kunming Mice | In vivo | 62.5-250mg/kg/d | 8d | Positive control ginkgo biloba tablet |
| 2003 | Efforts of *Cistanche deserticola* polysaccharides on the nitric oxids and apoptosis of lung in aging mice | Anti-aging | Polysaccharides | ICR Mice | In vivo | 25-100mg/kg | 42d | Blank control 0.9% saline |
| 2003 | Effects of *Cistanche* on apoptosis dopaminergic neurons induced by neurotoxin 1-methyl-4-phenylpyridinium | Antiapoptotic | Freeze-dried powder | New Zealand rabbits | In vivo | 0.8-3.2g/kg | 3d | Blank control 0.9% saline |
| 2003 | Protective effects of glycosides of *Cistanche* on cerebral ischemia in awaken mice | Protecting of cerebral ischemia | Cistanche glycosides | NIH Mice | In vivo | 62.5-250mL/kg | 8d | Positive control ginkgo leaf capsule |
| 2003 | protective effect of glycosides of *Cistanche* on T lymphocyte function of ^60^Coγ irradiated mice | Enhancing  immunity | Cistanche glycosides | NIH Mice | In vivo | 31.25-125mL/kg | 14d | Positive control astragalus injection |
| 2003 | The protective effect of glycosides of *Cistanche* against doxorubicin-induced cardiotoxicity in mice | Protect ischemic myocardium | Cistanche glycosides | NIH Mice | In vivo | 62.5-250mg/kg | 48h | Positive control vitamin E |
| 2003 | The protective effect of the glycosides of *Cistanche* on the cerebral hypoxia in mice | Protecting of cerebral hypoxia | Cistanche glycosides | NIH Mice | In vivo | 62.5-251mg/kg | 7d | Blank control 0.9% saline |
| 2003 | Effect of Rhubarb and *Cistanche deserticola* on lung injury rats of septic shock model | Protect of lung injury | Water  decoction | Sprague-Dawley rats | In vivo | 1-3g/kg | 15d | Blank control 0.9% saline |
| 2003 | Study on dosage effect relationship of laxative action of galactiol from *Cistanche deserticola* | Loosening bowel to relieve constipation | Galactitol | Kunming Mice | In vivo | 0.5mLl/ mouse |  |  |
| 2003 | Laxative action of separated parts from Herba *Cistanches* | Loosening bowel to relieve constipation | Galactitol | Kunming Mice | In vivo | 0.6mL/ mouse |  |  |
| 2004 | Neuroprotective effects of phenylethanoid glycosides from *Cistanches salsa* against 1-methyl-4-phenyl-1,2,3,6-tetrahydropyridine (MPTP)-induced dopaminergic toxicity in C57 mice | Neuroprotective effect | Phenylethanoid glycosides | C57mice | In vivo | 10-50mg/kg | 4d |  |
| 2004 | Stereolal study of protective effect of glycosides of *Cistanche* on mitochondria of cardiac muscle against adriamycin | Protect myocardial cell mitochondria | Cistanche glycosides | NIH Mice | In vivo | 62.5-250mg/kg | 6d | Blank control 0.9% saline |
| 2004 | Effects of glycosides of *Cistanche* on focal cerebral ischemic rats | Protecting of cerebral ischemia | Cistanche glycosides | Wistar rats | In vivo | 125-250mg/kg | 24h | Positive control ginkgo biloba tablet |
| 2004 | Effects of *Cistanche* on apoptosis of PC12 cells | Antiapoptotic | Crude drug | New Zealand rabbits | In vivo | 1.6-6.4g/kg | 24h |  |
| 2004 | Protective effects of water extract of desertliving *Cistanche* on oxidative damage of liver itochondria in aging model rat induced by D-gal | Anti-aging | Water  decoction | Wistar rats | In vivo | 2g/kg | 42d | Blank control 0.9% saline |
| 2004 | Influential of *C. deserticola* Y. C. Ma to acute lung injury in rats following septic shock | Protect of lung injury |  | Sprague-Dawley rats | In vivo | 200mg/kg | 15d | Blank control 0.9% saline |
| 2004 | Effects of *Cistanche deserticola* on immune function in aging model rat induced by D-gal | Anti-aging | Cistanche glycosides | Kunming Mice | In vivo | 125mg/kg/d | 15d | Blank control 0.9% saline |
| 2005 | Influence of glycosides of *Cistanche* on the ultrastructure of hippocampus of the D-galactose induced brain aging model mice | Anti-aging, Antioxidant | Cistanche glycosides | NIH Mice | In vivo | 31-125mg/kg/d | 50d | Positive control vitamin E |
| 2005 | The protective effects of *C. deserticola* Y. C. Ma on thymocytes in septic rats | Enhancing  immunity | Water  decoction | Wistar rats | In vivo | 1.25g/kg | 15d | Blank control 0.9% saline |
| 2005 | Studies on effect of *C. deserticola* Y. C. Ma on the function of liver mitochondria following septic shock | The impact on the liver mitochondria | Water  decoction | Sprague-Dawley rats | In vivo | 1000g/L | 15d | Blank control 0.9% saline |
| 2006 | Determination of acteoside in *Cistanche deserticola* and Boschniakia rossica and its pharmacokinetics in freely-moving rats using LC-MS/MS |  | Acteoside, Acteoside | Sprague-Dawley rats | In vivo | 10mg/kg | 24h |  |
| 2007 | Echinacoside prevents the striatal extracellular levels of monoamine neurotransmitters from diminution in 6-hydroxydopamine lesion rats | Neuroprotective effect | Echinacoside | Wistar rats | In vivo | 3.5-7.0 mg/kg | 7d | Blank control 0.9% saline |
| 2007 | Neuroprotective effects of echinacoside in the mouse MPTP model of Parkinson's disease | Neuroprotective effect | Echinacoside | C57BL/6 mice | In vivo | 5-20mg/kg | 15d | positive control amantadine |
| 2007 | Experiment study of desertliving *Cistanche* polysaccharides on regulated haematogenesis in bone marrow depressed anemia mice | Protecting the hematopoietic system | Polysaccharides | BALB/c Mice | In vivo | 50-100mg/kg | 7d | Blank control 0.9% saline |
| 2007 | Study on antifatigue effect of caulis *Cistanche* on the mice with Yang asthenia | Anti-fatigue | Water  decoction | Kunming Mice | In vivo | 2.5-10g/kg | 10d | Blank control 0.9% saline |
| 2007 | Effects of glycosides of *Cistanche* on learning and memory function in rat undertaken bilateral common carotid artery ligation and its possible mechanism | Improving cognitive functioning | Cistanche glycosides | Sprague-Dawley rats | In vivo | 40-160mg/kg | 14d | Positive control ginkgo biloba tablet |
| 2007 | Effect of water extract of Herba *Cistanchis* on NO level in ectogenesis embryo of pig | Promoting cell  growth and  development, | Water  decoction | Pig oocyte | In vitro | 0.05µg | 144h | Blank control 0.9% saline |
| 2007 | Effect of *C. deserticola* Y. C. Ma on energy metabolic function of liver mitochondria following sepsis | The impact on the liver mitochondria | Water  decoction | Sprague-Dawley rats | In vivo | 200mg/kg | 15d | Blank control 0.9% saline |
| 2007 | Effect of *C. deserticola* Y. C. Ma on protecting liver tissues of load motion mice | Hepatoprotection | Water  decoction | Kunming Mice | In vivo | 3g/kg | 16d | Blank control 0.9% saline |
| 2008 | Herba *Cistanche* extract enhances mitochondrial ATP generation in rat hearts and H9C2 cells | Enhances mitochondrial ATP generation in rat hearts and H9C2 cells | Methanol extract | rat | In vitro |  |  |  |
| 2008 | Protective effects of Herba *Cistanches* against the neurotoxicity of amyloid peptide in SH-SY5Y cells | Resistance to neurotoxicity | Aqueous extract | Brain cell SH-SY5Y | In vitro | 0.1-1mg/L | 48h |  |
| 2008 | Effect of Roucongrong on thymus cell apoptosis in rats with septicaemia | Antiapoptotic | Water  decoction | Wistar rats | In vivo | 1.25g/kg | 24h | Blank control 0.9% saline |
| 2008 | Effect of *Cistanche deserticola* on the expression of NOS3 with load movement in mice liver | Anti-fatigue | Water  decoction | Kunming Mice | In vivo | 0.2g/d | 15d | Blank control 0.9% saline |
| 2008 | Effective of *C. deserticola* Y. C. Ma to rats hepatocytic mitochondrial ATP ase following septic shock | The impact on the liver mitochondria | Water  decoction | Sprague-Dawley rats | In vivo | 1g/mL | 15d | Blank control 0.9% saline |
| 2009 | Echinacoside Elicits Endothelium-Dependent Relaxation in Rat Aortic Rings via an NO-cGMP Pathway | Vasorelaxant activity | Echinacoside | Sprague-Dawley rats | In vivo | 30–300µM | 20min |  |
| 2009 | The Hypocholesterolemic Effects of *Cistanche tubulosa* Extract, a Chinese Traditional Crude Medicine, in Mice | Hypocholesterolemic effects | Ethanol extract |  |  |  |  |  |
| 2009 | Echinacoside retards cellular senescence of human fibroblastic cells MRC-5 | Anti-aging | Echinacoside |  | In vitro |  |  |  |
| 2009 | Effect of *Cistanche deserticola* on thymic function damage rats in lung liver | Hepatoprotection  腺损伤 | Water extract | Sprague-Dawley rats | In vivo | 200mg/kg | 15d | Blank control 0.9% saline |
| 2009 | Effects of glycosides of Tripterygium wilfordii and *Cistanche deserticola* on the fertility of male mice | Improving the reproductive system | Water  decoction | Kunming Mice | In vivo | 10-25g/kg/d | 20d |  |
| 2009 | Study on the antioxidant activies of phenylethanoid glycosides from *Cistanche deserticola* | Antioxidant | Phenylethanoid glycosides |  | In vitro |  |  |  |
| 2009 | Protective effect of *Cistanche* glycosides on ethanol-induced liver damage in mice | Hepatoprotection | Cistanche glycosides | Kunming Mice | In vivo | 50-100mg/kg | 28d | Blank control 0.9% saline |
| 2009 | Study of antifatigue effect of Herba *Cistanche* decoction on athletic mice | Anti-fatigue | Water  decoction | Kunming Mice | In vivo | 0.25-1.0g/mL | 30d | Blank control 0.9% saline |
| 2009 | The clinical research for the treatment of alzheimer’s disease of *Cistanche* glycosides deserticola | Anti-Alzheimer's Disease | Cistanche glycosides | Human | In vivo | 6 capsules/d | 84天 | Positive control Aricept |
| 2009 | The effect of *Cistanche deserticola* polysaccharides on marcrophages activation | Enhancing  immunity | Polysaccharides | BALB/c,C3H/Hej Mice | In vitro | 3.125-50.00mg/L | 24h |  |
| 2009 | Enhancing effect of polysaccharides of *C. deserticola* Y. C. Ma on lymphocyte proliferation | Enhancing  immunity | Polysaccharides | BALB/c mice | In vitro | 1.56-100mg/L | 68h | Blank control RPMI-1640 culture |
| 2009 | Study on the extraction technology and antimicrobial action of *Cistanche deserticola* polysaccharides | Anti-inflammatory | Polysaccharides |  | In vitro |  |  |  |
| 2009 | Structure-activity relationships of phenylethanoid glycosides in plants of *Cistanches salsa* on antioxidative activity | Antioxidant | Phenylethanoid glycosides |  | In vitro |  |  |  |
| 2010 | Antifatigue Activity of Phenylethanoid-rich Extract from *Cistanche deserticola* | Anti-fatigue | Phenylethanoid  rich extract, Phenylethanoid glycosides | ICR mice | In vivo | 0.25-1.0g/kg | 21d | Blank control 0.9% saline |
| 2010 | Acylated phenylethanoid oligoglycosides with hepatoprotective activity from the desert plant *Cistanche tubulosa* | Hepatoprotection  activity | Methanolic extract | ddY mice | In vivo | 250-1000mg/kg |  |  |
| 2010 | Inhibitory Effect of Acteoside Isolated from *Cistanche tubulosa* on Chemical Mediator Release and Inflammatory Cytokine Production by RBL-2H3 and KU812 Cells | Antiallergic activity | Acteoside | RBL-2H3 cells | In vitro | 0.1-100.0 µg/mL | 24h |  |
| 2010 | Experimental study of directional differentiation of bone mesenchymal stem cells to osteoblasts guided by serum containing *Cistanche deserticola* | Induce  osteoblastic differentiation | Drug-containing serum | (bone mesenchymal stem cells,BMSCs) | In vitro | 10% drug-containing serum | 20d | Positive control dexamethasone |
| 2010 | Hepatoprotective effect and antioxidant activity of cultivate *C. deserticola* Y. C. Ma | Hepatoprotection  Antioxidant | Different solvent  extracts | Kunming Mice | In vivo | 250-1000mg/kg | 2h | Positive control Bifendate Pills, Dangfei Liganning capsule |
| 2010 | Protective effect of *Cistanche* phenylethanoid glycosides on sperm oxidative damage in rats in vitro | Antioxidant | Phenylethanoid glycosides | Wistar rats | In vivo | 0.025-0.5g/mL |  | Positive control vitamin C +FeSO_4_/H_2_O_2_ |
| 2010 | Influences of Roucongrong on hematopoietic function and immune function in tumor-bearing mice after chemotherapy | Enhancing  immunity | Aqueous extract | Kunming Mice | In vivo | 5-10g/kg | 10d | Blank control 0.9% saline |
| 2010 | Protective effect of glycosides of *Cistanche* on CCl_4_ damage mice | Hepatoprotection | Cistanche glycosides | Kunming Mice | In vivo | 62.5-125mg/kg/d | 24h | Blank control 0.9% saline |
| 2010 | Clinical observation on the improvement in hemodialysis adequacy of *Cistanche deserticola* | Kidney protection | Water  decoction | Human | In vivo | 20g | 24h |  |
| 2010 | The inhibitory effects of phenylethanoid glycosides in *Cistanche* on the activity of tyrosinase | Tyrosinase activity inhibition | Phenylethanoid glycosides |  | In vitro |  |  |  |
| 2010 | Effects of *Cistanche deserticola* on acute lung injury in rats with sepsis | Protect of lung injury |  | Sprague-Dawley rats | In vivo | 1-5g/mL | 15d | Blank control 0.9% saline |
| 2010 | Effects of glycosides of *Cistanche* on excitatory amino acid content in brain tissue of rats with cerebral ischemia and reperfusion | Protecting cerebral ischemia | Cistanche glycosides | Sprague-Dawley rats | In vivo | 4.0g/kg | 2d | Blank control 0.9% saline |
| 2011 | *Cistanches* Herba enhances learning and memory by inducing nerve growth factor | Memory  improvement | Cistanches Herba extract |  | In vitro | 10-250 µg/mL | 48h | [Positive control nerve growth factor50 ng/ml](http://www.sciencedirect.com.ezproxy.uct.ac.za/science/article/pii/S0166432810006315#200017366) |
| 2011 | Effects of *Cistanche deserticola* on behavior and signs of cataract and retinopathy in senescence-accelerated OXYS rats | Vasorelaxant activity, Neuroprotective effect | Aqueous extract | OXYS and Wistar rats | In vivo | 15mg/kg |  |  |
| 2011 | Water-soluble carbohydrate compound from the bodies of Herba *Cistanches*: Isolation and its scavenging effect on free radical in skin | Antioxidant | Water  decoction | SD rats | In vivo | 0.3-0.7% | 47d |  |
| 2011 | Effects of Herba *Cistanches* on the ability of resistance exercise-induced fatigue in rats and free radical in brain tissue | Anti-fatigue |  | Wistar rats | In vivo |  |  | Blank control 0.9% saline |
| 2011 | Effects of *Cistanche* total glycosides on promoting olfactory ensheathing cells’ proliferation and glial-cell-line-derived neurotrophic factor secretion | Neuroprotective effect |  | Wistar rats | In vivo | 1ug-100mg/mL | 24h | Negative control serum-containing medium |
| 2011 | Protective effect of *Cistanche* glycosides on ethanol-induced damage in the primary hepatocytes of mice | Hepatoprotection | Cistanche glycosides |  | In vitro | 0.2-0.8g/L | 24h | Blank control 0.9% saline |
| 2011 | Influences of Herba *Cistanche* tea on the ability of anti-fatigue and anoxia endurance in mice | Anti-fatigue, Antioxidant | Cistanche tea | NIH Mice | In vivo | 41.67-166.67mg/mL | 35d | Blank control distilled water |
| 2011 | The experimental research on toxicological safety of *Cistanche deserticola* tea | Toxicological test | Water extract | Kunming Mice,Wistar rats | In vivo | 3000-12000mg/kg | 90d | Blank control distilled water |
| 2012 | Echinacoside stimulates cell proliferation and prevents cell apoptosis in intestinal epithelial MODE-K cells by up-regulation of transforming growth factor-beta1 expression | Loosening bowel to relieve constipation | Echinacoside | MODE-K | In vitro |  |  |  |
| 2012 | *Cistanches* herba Induces Testis Cytotoxicity in Male Mice | Testis Cytotoxicity |  | ICR mice | In vivo | 250-1000 mg/kg | 35d |  |
| 2012 | *Cistanche deserticola* extract increases bone formation in osteoblasts | Increases bone formation in osteoblasts |  |  | In vitro |  |  |  |
| 2012 | Effect of Herba *Cistanches* on testosterone content, substance metabolism and exercise capacity in rats after exercise training | Anti-fatigue | Ethnol extract | Wistar rats | In vivo | 6.01g/kg/d | 49d | Blank control 0.9% saline |
| 2013 | *Cistanche deserticola* decoction alleviates the testicular toxicity induced by hydroxyurea in male mice | Testis Cytotoxicity | Crude drugs | Kunming mice | In vivo | 1.5-6.0g/kg | 28d |  |
| 2013 | Herba *Cistanches* stimulates cellular glutathione redox cycling by reactive oxygen species generated from mitochondrial respiration in H9c2 cardiomyocytes | Protect ischemic myocardium |  |  |  | 10-300 µg/mL |  |  |
| 2013 | Anti-hyperglycemic and hypolipidemic effects of *Cistanche tubulosa* in type 2 diabetic db/db mice | Regulate blood glucose and the  blood lipids |  | BKS.Cg-*Dock7^m^* +/+ *Lepr^db^*/J (*db*/*db*) mice | In vitro | 24.2-120.9mg/kg | 45d | Normal controls age matched *db*/+ mice |
| 2013 | Subchronic toxicity of *Cistanche Tubulosa* extract on SD rat | Toxicological test |  | Sprague-Dawley rats | In vivo | 2500-7500mg/kg | 90d | Blank control basic food |
| 2013 | The effect of *Cistanche deserticola* drug-containing serum on BMSC differentiation into neural cells | Induction of neuronal progenitor cell growth | Water  decoction | Sprague-Dawley rats | In vivo | 33g/kg | 10d | Positive control β-mercaptoethanol 1mmol/L |
| 2013 | Effect of polysaccharides of *Cistanche deserticola* on learning and memory ability and oxygen stress of model rats with Alzheimer’s disease | Anti-Alzheimer's Disease | Polysaccharides | Wistar rats | In vivo | 50-200mg/kg/d | 7d | Normal controls corn oil |
| 2014 | Amelioration of dextran sulphate sodium-induced colitis in mice by echinacoside-enriched extract of *Cistanche tubulosa* | Loosening bowel to relieve constipation | Echinacoside | C57BL/6Jmice | In vivo | 20mg/kg/d | 2d | Blank control DSS drinking water |
| 2014 | Effect of Roucongrong (Herba *Cistanches Deserticolae*) on reproductive toxicity in mice induced by glycoside of Leigongteng (Radix et Rhizoma Tripterygii) | Reproductive  toxicity | Crude drugs | BALB/c mice | In vivo | 10-30mg/kg | 21d | Normal controls glycoside extracted from Leigongteng |
| 2014 | Echinacoside ameliorates D-galactosamine plus lipopolysaccharide-induced acute liver injury in mice via inhibition of apoptosis and inflammation | Hepatoprotection | Echinacoside |  | In vivo | 60 mg/kg | 1h |  |
| 2014 | Protective Activities of Cistanoside A on Alcohol Induced Hepatotoxicity in Mice | Hepatoprotection | Cistanoside A |  | In vivo | 10-20mg/kg/d | 14d |  |
| 2014 | Acylated phenylethanoid glycosides, echinacoside and acteoside from *Cistanche tubulosa*, improve glucose tolerance in mice | Improving glucose tolerance |  | ddY mice | In vivo | 125-500 mg/kg | 14d |  |
| 2014 | Reversal by aqueous extracts of *Cistanche tubulosa* from behavioral deficits in Alzheimer's disease-like rat model: relevance for amyloid deposition and central neurotransmitter function | Anti-Alzheimer's Disease | Phenylpropanoid glycosides | Sprague-Dawley rats | In vivo | 100-200mg/kg | 15d |  |
| 2014 | Extracts of *Cistanche deserticola* Can Antagonize Immunosenescence and Extend Life Span in Senescence-Accelerated Mouse Prone 8 (SAM-P8) Mice | Anti-aging |  |  | In vivo | 100-2500mg/kg | 28d |  |
| 2014 | The protection on acute liver injury in mice of *Cistanche deserticola* | Hepatoprotection | Ethnol extract | Kunming Mice | In vivo | 0.02-0.18g | 6d | Positive control Yiganling mixed suspension |
| 2014 | Study on toxicity of *Cistanche deserticola* | Toxicological test | Cistanche powder | ICR Mice, Sprague-Dawley rats | In vivo | 2.5-20g/kg | 14d | Positive control Cyclophosphamide |
| 2014 | Effect of phenylethanoid glycosides from *Cistanches* Herba hemodynamics on rats with high altitude pulmonary hypertension | Protect of lung injury | Phenylethanoid glycosides | Wistar rats | In vivo | 75-300mg/kg/d | 28d | Blank control 0.9% saline |
| 2014 | Therapeutic effect of phenylethanoid glycosides on cyclopfosphamide-induced dyszoospermia in mice and its mechanism | Improving the reproductive system | Phenylethanoid glycosides | BALB/C Mice | In vivo | 50-100mg/kg | 30d | Blank control 0.9% saline |
| 2015 | Echinacoside Protects against 6-Hydroxydopamine-Induced Mitochondrial Dysfunction and Inflammatory Responses in PC12 Cells via Reducing ROS Production | Anti-inflammatory | Echinacoside | PC12 Cells | In vitro | 0.1-10µM |  |  |
| 2015 | Glycosides of *Cistanche* improve learning and memory in the rat model of vascular dementia | Improve learning and memory | Cistanche glycosides | Wistar rats | In vivo | 10-450mg/kg | 14d |  |
| 2015 | Evaluation of the Intestinal Transport of a Phenylethanoid Glycoside-Rich Extract from *Cistanche deserticola* across the Caco-2 Cell Monolayer Model | Loosening bowel to relieve constipation | Phenylethanoid glycosides | Caco-2 cell | In vitro |  |  |  |
| 2015 | HPLC/Q‐TOF‐MS‐Based Identification of Absorbed Constituents and Their Metabolites in Rat Serum and Urine after Oral Administration of *Cistanche deserticola* Extract | Absorption substance | Ethanol extract | Wistar rats | In vivo | 10mL/kg | 3d |  |
| 2015 | *Cistanche tubulosa* ethanol extract mediates rat sex hormone levels by induction of testicular steroidgenic enzymes | Raise the level of sex hormones | Ethnol extract |  |  |  |  |  |
| 2015 | Preventive effects of phenylethanol glycosides from *Cistanche tubulosa* on bovine serum albumin-induced hepatic fibrosis in rats | Hepatoprotection |  | Sprague-Dawley rats |  |  |  |  |
| 2015 | Study on laxative constitutes in *C. deserticola* Y. C. Ma | Loosening bowel to relieve constipation | Different solvent extracts | ICR Mice | In vivo |  | 6h | Blank control distilled water |
| 2015 | Cerebral protective effect of *Cistanche* in Alzheimer’s patients | Anti-Alzheimer's Diseas | Capsules | Human | In vivo | 0.9g/d | 336d | Positive control Aricept |
| 2015 | Toxicological security evaluation of herba *Cistanches* granule | Toxicological test |  | Kunming Mice | In vivo | 1875-15000mg/kg | 30d | Blank control basic food |
| 2015 | Protective effect of phenylethanoid glycosides from *Cistanche tubulosa* on rats with high aititude cerebral edema | The impact on the brain edema | Phenylethanoid glycosides | Wistar rats | In vivo | 75-300mg/kg/d | 10d | Positive control Rhodiola Oral Solution |
| 2015 | Protective effect of total glycosides from *Cistanche salsa* on experimental liver injury | Hepatoprotection | Cistanche glycosides | Kunming Mice | In vivo | 65-130mg/kg | 10d | Positive control Bifendate Pills |
| 2015 | Efficacy research of Cynomorium and *Cistanche* mistures | Anti-fatigue |  | Kunming Mice | In vivo | 0.15-0.3mg/g | 7d |  |
| 2015 | Experimental research about the effect of glycosides on cognitive function and tau, P-tau, Aβamyloid protein expression in hippocampal brain areas of vascular dementia rats model | Improving cognitive functioning | Cistanche glycosides | Wistar rats | In vivo | 205-10mg/kg/d | 14d | Positive control Oxiracetam 450mg/kg/d |
| 2015 | Protective effect of *Cistanche* total glycosides pretreatment against renal ischemia reperfusion injury in rats | Kidney protection | Cistanche glycosides | Sprague-Dawley rats | In vivo | 400mg/kg |  |  |
